# COVID-19: Critical discussion on the applications and implications of chemicals in sanitizers and disinfectants

**DOI:** 10.17179/excli2020-1386

**Published:** 2020-06-15

**Authors:** Olubunmi Atolani, Mariam Temitope Baker, Oluyomi Stephen Adeyemi, Ismaeel Ridwan Olanrewaju, Abdulmumeen A. Hamid, Oloduowo M. Ameen, Stephen O. Oguntoye, Lamidi A. Usman

**Affiliations:** 1 Department of Chemistry, University of Ilorin, P.M.B. 1515, Ilorin, Nigeria; 2 Department of Biochemistry, Medicinal Biochemistry, Infectious Diseases, Nanomedicine & Toxicology Laboratory, Landmark University, PMB 1001, Omu-Aran 251101, Kwara State, Nigeria

## ⁯

***Dear Editor, ***

The anxiety and trauma associated with the tragic coronavirus disease pandemic coded, COVID-19 led many to indulge in various unorthodox preventive measures such as the extensive indiscriminate use of alcohol-based hand sanitizers (ABHS), abuse, misuse, overdose of prescription drugs like chloroquine, hydroxychloroquine and chloroquine phosphate globally. While some preventive measures are recommended and adopted, such as national lockdown, self-isolation, quarantine, stay-at-home model, avoidance of large gathering, social distancing, wearing of face-masks and hand gloves, periodic hand washing particularly with liquid soaps/detergents under running tap water, avoidance of touching the face among others, the use of ABHS has been more prominent. ABHS contains on average 60-70 % by weight of one or more alcohols. During the 2019/2020 COVID-19 pandemic, the use of ABHS was more renowned to the extent that some individuals recommended the application on the hands every 30 minutes for a period of at least 20 seconds while outside the home. Though, the periodic application of the hand sanitizers seems like an effective on-the-go solution to preventing the spread of the virus, many other associated hazards call for caution. Besides transdermal absorption leading to mortality and morbidity of varying degree; alcohol-alcohol adulteration, deliberate and unintentional ingestion of ABHS may result in respiratory depression, irreversible blindness, intoxication, cirrhosis, acidosis, headache, central nervous system depression, seizure, hypoglycemia, coma, or even death in some cases. The non-ABHS are equally not absolutely safe as many of them contain active agents that are allegedly carcinogenic, toxic, inducing microbial resistance and endocrine disruption. Considered together, this implies that while some may not die from contracting the disease, the preventive measures taken could lead to death or other forms of morbidity thereby revealing that there is indeed, death in preventive measures when done without cautionary measures. This study seeks to highlight some associated risks in the use of ABHS and non-ABHS, whilst advocating the use of safer or 'greener' alternative procedure for use as preventive measures particularly during the COVID-19 pandemic. 

The sudden outbreak of the coronavirus disease epidemic preferably referred to as COVID-19 was a tragic global pandemic that claimed over 190,000 lives with over two million reported cases spreading across 210 countries within the first five months (December, 2019 - 24^th^ April, 2020) of the outbreak across the globe (Worldometers, 2020[102]). While the highest toll of mortality remained in China, Italy, Spain and America as at the end of March, 2020, many Asian and tropical African countries such as Iran, South Africa, Algeria, Egypt, Nigeria and Cameroon had got their acrid taste of the pandemic. The fear, anxiety and emotional trauma associated with the outbreak of COVID-19 which started from the Wuhan Province in China in December 2019 was quite rattling, monumental and terrific as many erroneously regarded contracting the infection as a death penalty. In fact, infected individuals who recovered from the disease are stigmatized and disenfranchised, segregated and avoided. The long and short term implications of contracting the disease made citizens of most countries to intensify efforts at preventing the COVID-19 disease. While global mortality rate rose to 3.4 % in some countries (Worldometers, 2020[102]), the trauma associated with the infection had no limit. The incident obviously led to improvement in hand hygiene (HH) in order to curtail the spread of the virus. This has brought to limelight the prominence of alcohol-based hand sanitizer, ABHS and other non-alcohol based hand sanitizers as well as household disinfectants.

### Common preventive measures against the COVID-19 

As at this moment, there is no vaccine for the prevention of COVID-19. The best prevention is to avoid being exposed to the virus (Adhikari et al., 2020[1]). As a result of this, government and individuals adopted various measures in preventing the spread of the disease. The government and health sector' advocacy groups contributed tremendously to the spike in the use of various preventive methods such as city/town and national lockdown, self-isolation, quarantine, stay-at-home model, avoidance of crowd, social distancing, wearing of face-mask and hand gloves, periodic hand washing particularly with liquid soaps/detergents, use of ABHS, avoidance of touching the face among others. Following unsubstantiated claims, some individuals in the developing countries resulted to the overconsumption of acclaimed immune-boosting substances such as ginger, garlic, lemon fruits, and salt solution etc. Besides, some in the tropical countries believed that the hot weather could reduce the chances of survival of the virus, hence, they resulted to staying long hours in the bright day sunlight and/or periodic consumption of hot drinks such as water, tea and juice with the intention of providing an unhealthy high temperature environment for the survival of the infectious virus. Some other people resulted to the consumption of food or substances that increases the pH of the body to the alkaline region since there were unsubstantiated claims that the virus does not survive at alkalinic pH. Although viruses are sensitive to pH and temperature changes and cannot survive beyond a narrow range (Sturman et al., 1990[85]; Mi et al., 2019[62]), a crude means of achieving such could be more deadly than envisaged.

Moreover, the tentative approval by the United States tentative of chloroquine-based therapy as trial for the management of COVID-19 patients on compassionate ground (based on WHO guideline), led some individuals to an unethical practice of the indiscriminate consumption of chloroquine-based drugs as both prophylaxis and curative. Although, initial trials indicated some interesting positive results at minute doses (Gao et al., 2020[33]; Liu et al., 2020[54]; Singh et al., 2020[82]), reports are still premature and yet to be universally accepted. If clinical data confirm the antiviral activity of chloroquine-based drugs, the novel COVID-19 could become one of the cheapest and simplest to treat among infectious respiratory diseases. This is because the drugs are quite affordable (Raoult et al., 2020[73]). The affordability and accessibility of the chloroquine-based drugs partly led to the abuse with resultant lethality and health hazards which included abdominal discomfort, coma and even death in some cases (Wong et al., 2011[101]). In fact, the moderate dose of chloroquine can cause effects such as depression, psychosis, delirium, mood swing, personality change among others (Good and Shader, 1982[36]). The close derivative, hydroxychloroquine is also known to possess side effects such as retinal toxicity, cardiovascular toxicity, refractory shock and ventricular arrhythmias (Fung et al., 2007[32]; Rüther et al., 2007[76]; Marmor et al., 2016[60]; Tsang et al, 2019[91]). Meanwhile, chloroquine phosphate and a few other derivatives have been reported as potential anti-dote to COVID-19 infection (Gao et al., 2020[33]; Sahraei et al., 2020[78]).

Although, many of these preventive measures were often adopted, many of them frequently present mild to severe implications with only a few of them proving really potent and safe. In reality, some of the measures were quite detrimental to human health and it could result to death before even contracting the dreaded viral infection, COVID-19 (Choi et al., 2017[22]; Chan and Chan, 2018[20]). While all the measures adopted thus far have both their merits and demerits, this report focuses more on the chemistry of the death and the associated danger, particularly in the use of hand sanitizers and other applied preventive chemicals against the spread of COVID-19 as well as other germs. 

### Chemical compositions of household products for preventive measures against COVID-19

The advent of the COVID-19 outbreak marked the commencement of the surge in the series of industrial and domestic-made chemicals like the hand sanitizers, liquid soaps, disinfectants and others. The upsurge in the global consumption of hand sanitizers following the COVID-19 pandemic was unprecedentedly geometrical. The use of hand sanitizer became well renowned to the extent that some individuals apply on their hands every 30 minutes especially when outside the home. While the practice seems a plausibly effective on-the-go solution in the prevention of the spread of the viral infection, other serious dangers are quite associated. Although, microorganisms pervade almost everywhere human survives (Bhoonderowa et al., 2014[10]; Kõljalg et al., 2017[47]) and the use of sanitizers can help in minimizing their spread and deleterious effect on human, the frequent use may however pre-dispose users to some degree of fatality (Chan et al., 2017[21]; Chan and Chan, 2018[20]).

Various sub-standard brands of hand sanitizers flooded both offline and online markets in the developed and developing countries. Besides the hike in the prices of these household products, their acquisition could be tantamount to self-purchase of quicker “death”. While the chemical composition of the various household antiviral preventive products used is sometimes regional or country-based, some chemicals are more accessible and universal to all. Some of them are thus discussed herein.

### Hand sanitizers and disinfecting soaps

Hand sanitizers are hygienic products that are applied on the hands to prevent the spread of microorganisms like viruses, bacterial and fungi such as coronaviruses, rhinoviruses, influenza viruses, herpes simplex virus, ebola virus, *Staphylococcus aureus, Enterococcus faecalis, Escherichia coli, *traveller diarrhoea among others, especially where access to water is limited (Fendler et al., 2002[30]; Conover and Gibson, 2016[24]; Wolfe et al., 2017[100]; Thaddeus et al., 2018[87]; Kuenzli et al., 2019[50]). Hand sanitizers, though, originally developed for the healthcare sector (Ascenzi, 1995[3]; Block, 2001[12]), it has become a routine for many people to carry hand sanitizer with them at all times, especially during the recent disease outbreak. 

**Figure 1:**
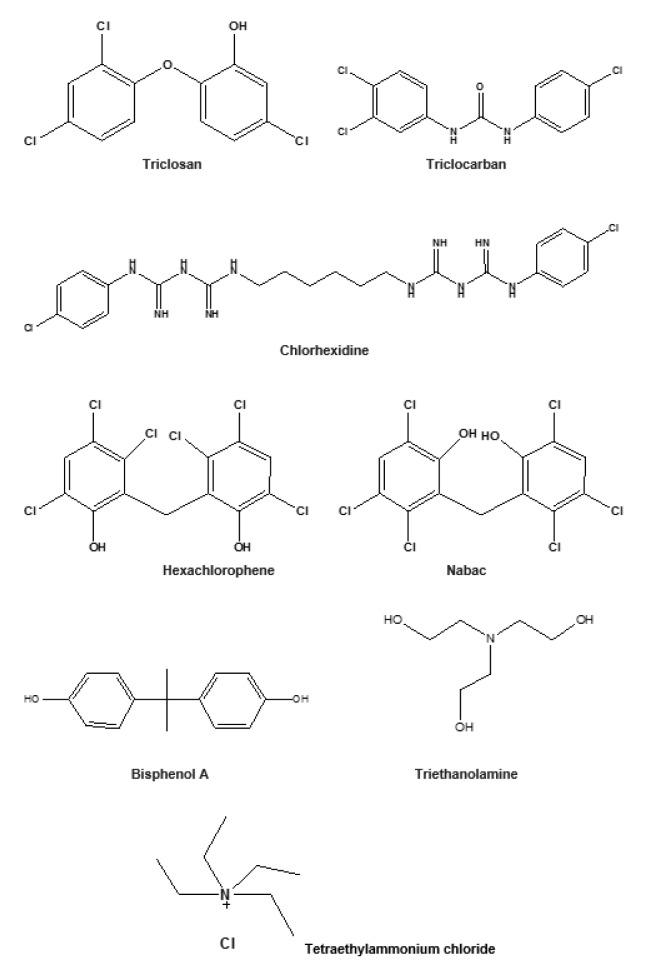
Some active agents used in sanitizers and disinfectants

Industrial and home-made hand sanitizers are made of various chemical compositions subject to what is locally available. A brief check of brands on the internet, local markets and the literatures indicated two main categories of sanitizers; alcohol-based and non-alcohol-based hand sanitizers (NABHS). The alcohol-based hand sanitizers (ABHS) are primarily composed of alcohols such as either ethanol, isopropyl alcohol, propan-1-ol or propan-2-ol; and additives such as colorants, stabilizers, fragrance and sometimes preservatives which may include formaldehyde, parabens among other chemicals (Luby et al., 2004[57], 2005[56]; Kramer et al., 2007[48]; Napolitani et al., 2020[65]). Other chemicals usually added for various purposes include polyacrylic acid, glycerin, carbomer, propylene glycol, triethanolamine (trolamine), tetraethylammonium chloride, dichlorodimethylphenol (Figure 1(Fig. 1)), chlorine, deionized water, and extract of plants (Wolfe et al., 2017[100]; Thaddeus et al., 2018[87]; Surini et al., 2018[86]). The World Health Organization guideline for the preparation of ABHS revealed the composition to be 80 % ethanol, 1.45 % glycerol and 0.125 % hydrogen peroxide or 75 % isopropyl alcohol, 1.45 % glycerol and 0.125 % hydrogen peroxide (WHO, 2010[97]). On the other hand, the non-alcohol based hand sanitizers (NABHS) are made up of various chemicals similar to the component found in the alcohol-based type with the exclusion of alcohol. However, anti-microbial or disinfecting agents such as triclosan (TCS), triclocarban, sodium hypochlorite, chlorhexidine, benzalkonium chloride and benzithonium chloride are often added (Hayat and Munnawar, 2016[42]; Bondurant et al., 2019[13]).

Some of the chemicals used in various disinfectants include chloroxyphenol B, oleum aromaticum, triclosan, triethanolamine, tetraethylammonium, dichlorodimethylphenol, chlorine, etc. It is quite imperative that antimicrobial agents, disinfectants and antiseptics substances possess strong activities against the pathogens including the biofilm-forming pathogens, else antimicrobial resistance could easily develop (Bridier et al., 2011[16]; Hauser et al., 2016[41]; Ghanem et al., 2018[35]). The assumption has led to the use of extremely strong and sometimes hazardous chemicals as disinfectants. For effectiveness or improved potency, combinations of two or more active ingredients are often used in some anti-viral disinfectants (Ahmed et al., 2020[2]).

A newly developed NABHS which contains only 14 % alcohol by volume and usually applied in small volume was reported to work synergistically with the other active agent in the sanitizer via a system called “quad system technology” to kill the virus and other germs (Cavage, 2010[17]).

Generally, in ABHS, formulations with ethanol between 60 and 95 % are known to be significantly effective in preventing spread of microbial infections (Kramer et al., 2002[49]) and are generally regarded safe (FDA, 1994[29]). WHO recommends washing of hands under running tap water, and when the resources for that is not available, ABHS comes handy. However, the ABHS are recommended over the use of soap due to its ease of use and capability in stalling microbial spread (WHO 2009[98]; Hayat and Munnawar, 2016[42]). However, some other potential alternatives such as nonorganic antibiotics and organic or natural biocides have been proposed in place of triclosan due to some of the reported side effects (Kim et al., 2007[46]).

### Associated hazards with ABHS for the prevention of COVID-19

Many ABHS carry a lot of risks. First, most ABHS contain alcohols in excess of 60 % which made them to be classified as Class I Flammable Liquid substance as a result of their flash point below 100 °C Fahrenheit (Kramer et al., 2002[49]; FDA, 2020[28]). Precisely, ethanol 80 % (v/v) and isopropyl alcohol 75 % (v/v) have flash points of 17.5 °C and 19 °C, respectively (WHO, 2020[95]). This quite indicates that the products can ignite at relatively low temperatures. 

**Figure 2:**
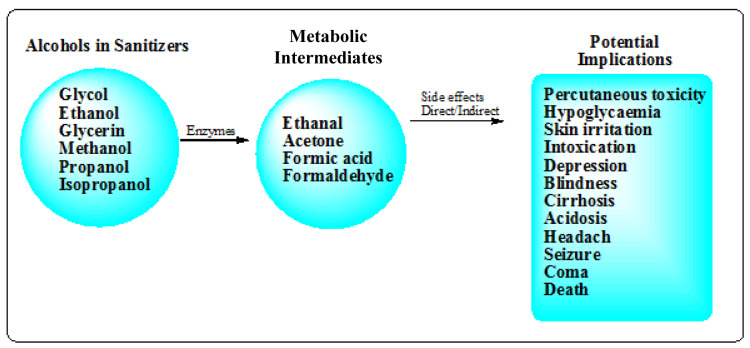
Potential health hazards of ingested or skin-absorbed alcohols in ABHS

Hence, ABHS increase the risk of fire accidents. Although, ABHS are a more accessible and usually a preferred choice for many people during epidemics like the COVID-19 compared to choices like the towelettes (Rai et al., 2017[72]), yet they carry their own significant risks. The risk associated with the use of the ABHS includes accidental poisoning via ingestion, fire hazard, organ toxicity via absorption through the skin among others (Figure 2(Fig. 2)). Children are often attracted to the nature, smell and cool feel of alcohol on the skin and series of accidental poisonings, including fatal ones in child ren have been reported (Chan and Chan, 2018[20]). For instance, investigation carried out on young children who swallowed ABHS revealed that they were diagnosed of apnea, acidosis and some even went into coma (Rayar and Ratnapalan*, *2013[74]). It has been established that younger children have decreased liver glycogen stores, which increases their risk of developing hypoglycemia and have various pharmacokinetic factors, which make them more susceptible to developing toxicity from alcohol (Tran et al., 2007[89]; Marek and Kraft, 2014[59]). 

In addition, alcohol-alcohol dilution is a major contamination in the industry. A more affordable alcohol with similar physical properties is sometimes used to adulterate another alcohol for pecuniary profit. If the ethanol for instance, is adulterated with other toxic alcohols such as methanol, irreversible blindness, coma or even death could result (Gormley et al., 2012[37]; Forrester, 2015[31]; Moore, 2019[63]). Methanol is known to be more lethal than ethanol as unintentional ingestion by children and deliberate consumption by older subjects often lead to poisoning requiring prompt antidotal therapy, critical care in addition to other supporting therapy (Chan and Chan, 2018[20]). Methanol is reportedly more toxic when inhaled, or exposed to human orally or through the skin. Alcohol concentration in the blood could increase via absorption through the skin when used frequently. Due to its toxicity, it is therefore strongly not recommended for use in hand sanitizers. However, some reports indicated that such contaminations have been observed in ABHS (Mowry et al., 2015[64]; Chan and Chan, 2018[20]). Transdermal poisoning resulting from skin absorption of methanol is well reported (Qiao and Guo, 1992[71]; Ryu et al., 2016[77]). In fact, exposure to methanol was reported to cause multiple sclerosis (Henzi, 1984[43]). Other types of alcohol-alcohol intoxication and poisoning have been reported (Chan et al., 2017[21]; Chan and Chan, 2018[20]).

The commonly used alcohol in ABHS, ethanol, is a known toxicant and central nervous (CNS) depressant capable of inducing hypoglycemia (Baum 2017[9]; Moore, 2019[63]). The use of isopropanol-based hand sanitizers carries equal or even greater risk than ethanol-based counterparts. Isopropanol besides being intoxicating, it is converted in the liver to acetone (propanone) which causes greater CNS depression than ethanol (Litovitz, 1986[53]). Various deaths and morbidities associated with ABHS have been reported (Gormley et al., 2012[37]; Darracq et al., 2013[25]; Jones et al., 2013[45]; Forrester, 2015[31]; Basyal et al., 2018[8]; Moore, 2019[63]). Interestingly, there has been emerging cases of alcohol-tolerant microorganisms which therefore cast aspersion on the future of the use of alcohol in hand sanitizers (Pidot et al., 2018[70]; Bondurant et al., 2019[14]). It is predicted that more organisms would develop resistance to alcohol in the near future if indiscriminate discharge to the environment is not controlled.

Other adverse health effects that could result from both ABHS and NABHS include ocular irritation, vomiting, conjunctivitis, oral irritation, cough, and abdominal pain (Qiao and Guo, 1992[71]; Choi et al., 2017[22]; Chan and Chan, 2018[20]). In some rare scenario, adverse effects could include acidosis, respiratory depression, headache, hypoglycemia, irreversible blindness, central nervous system depression, seizure, coma and death (Figure 2(Fig. 2)). These effects are noted to be more severe in ABHS (Bonner, 2017[15]; Choi et al., 2017[22]; Chan and Chan, 2018[20]). The continuous use or overuse of hand sanitizers can cause chronic irritation and severe skin breakdown among other effects (Qiao and Guo, 1992[71]), especially in children. A study that reported the incidences of poisoning associated with ABHS between 2011 and 2014 indicated that children below 12 years had incidences of over 70,000 exposures to hand sanitizers, of which 92 % are due to ABHS with only 8 % NABHS exposure (Bonner, 2017[15]).

Furthermore, the continuous topical application of ethanol on the skin was reported to lower skin barrier functions, thereby rending the membrane highly susceptible to harmful chemicals in soaps and cosmetics (Lachenmeier, 2008[51]). Moreso, percutaneous toxicity could also occur in children with lacerated skin (Lachenmeier, 2008[51]). In fact, the potential for abuse of ABHS has raised more concern now than before. Although, ABHS seem easier to use and proved higher efficiency than some detergent-based sanitizers, the repeated exposure of users to the ABHS calls for caution (Perez et al., 2019[69]).

### Environmental hazards of triclosan in hand sanitizers and liquid soaps

Triclosan (TCS: 2, 4, 4'-trichloro-2'-hydroxydiphenyl ether) is a universal antimicrobial agent that has found its way into many pharmaceuticals and personal care products as well as in the promotion of growth in animal including aquatic species (Daughton and Ternes, 1999[26]). It is used as antibacterial agent in many consumers' and household products such as medicated soaps, hand sanitizers, deodorants, toothpastes, air fresheners and other cosmetic products (Sethuraman et al., 2014[80]). It is currently being considered as an emerging contaminant (Wang et al., 2018[93]).

Triclosan with its related compound, triclocarban (Figure 1(Fig. 1)) used in consumer's products such as liquid soaps and hand sanitizers previous mentioned is of paramount concerns to scientists due to its long persistency in the environment (FDA, 2016[27]; USGS, 2016[92]). Its ability to cause disruption of the endocrine (hormone) system is still being evaluated and already recommended to be avoided (Bonner, 2017[15]; Stoker et al., 2010[84]). It was classed as a pesticide in the National Pesticide Information Retrieval System (NPIRS [Triclosan], 2020[67]) of the United States. Previously, hexachlorophene, an organochlorine compound known as Nabac (Figure 1(Fig. 1)) with structural similarity to triclosan had been banned as disinfectant by the FDA following health concerns due to the allegedly associated teratogenicity (IARC, 1998[44]; Halden, 2014[39]). TCS together with bisphenol A were reported to possess potential to weaken the immune system (Clayton et al., 2011[23]). TCS is reported to cause oxidative stress and induce genotoxicity in goldfish, suggesting a potential ecotoxicological risk to aquatic ecosystems (Stasinakis et al., 2008[83]; Halden, 2014[39]; Silva et al., 2015[81]). TCS has been reported as persistent organic pollutants that are retained for months or years in water bodies (Lygina et al., 2013[58]).

### Toxicity and side-effects of disinfectants, adulterated and sub-standard chemicals in soaps and hand sanitizers on the users

Antibiotics abuse, overuse and misuse of soaps and hand sanitizers is often responsible for effects such as antibiotics resistance and endocrine disruption (Atolani et al. 2016[7]; Lu et al., 2018[55]). TCS, a typical antibiotic is commonly used in many soap preparations and some hand sanitizers are known to produce high anti-microbial efficiency. However, many studies have implicated it as a source of induction of antibiotic resistant microorganisms as the use of only 0.2 mg/L TCS for only 30 days can cause multi-drug resistance to *Escherichia coli *and *Staphylococcus aureus *of which similar effects were not reported for chlorhexidine or hydrogen peroxide-based agents (Westgate et al., 2016[94]).

The use of alcohol in ABHS is equally not absolutely safe as it can easily be absorbed through the skin. Reports indicated that blood concentration of ethanol could reach 2.3 % within 90 minutes of use of ABHS (Kramer et al., 2007[48]). In the study, ethanol and acetaldehyde concentrations were as high as 1.7 and 1.95 mg/L respectively. Ethanol is often converted to acetaldehyde which is also absorbed into the blood stream (Thompson et al., 2005[88]; NPIRS [Ethanol] 2020[67]). The metabolic products are often more toxic in the human system. High concentration of ethanol in human blood ingested via consumption of alcoholic beverages is known to cause both short-term and long-term toxicity such as inebriation and liver cirrhosis. Hence, the use of ABHS could invariably lead to increased concentration of alcohol in human blood (Kramer et al., 2007[48]). 

There are increasing incidences of intentional ingestion of ethanol-containing hand sanitizers especially by alcohol addicts (Gormley et al., 2012[37]). Since children are attracted to the scent, bright coloration and the attractive packaging of ABHS, they are generally more at risk (Rayar and Ratnapalan, 2013[74]). Within 2011 - 2016, U.S. poison control centers reported over 100,000 calls with regards to children exposure to hand sanitizer (Georgia Poison Center, 2015[34]; CDC, 2020[18]).The intoxication by the ingestion of hand sanitizers is a major overlooked risk seeking urgent attention globally (Jones et al., 2013[45]; Raza et al., 2014[75]; Moore, 2019[63]). High degree of morbidity and mortality are risks associated with the use of ABHS (Santos et al., 2017[79]). Therefore, following the proliferation in the production of ABHS due to the COVID-19 pandemic, there is an obvious need for global caution and conscientiousness in the acquisition and utilization of hand sanitizers.

### Soaps and ABHS: efficiency versus safety potential

It was reported that most germs or microbial pathogens cannot survive adequate washing with liquid soaps. The structure of most pathogenic bacterial and virus is less resistant to the potency of the alkalinity, the corrosive nature and membrane-shattering ability of most liquid soaps. While the proper washing with the liquid soaps kills the pathogens up to 99.9 % the rinsing with clean water helps to eliminate the dead organisms from hands. Most liquid soaps are water-based; hence, this naturally reduces the skin penetrating effect of some of the components compared to ABHS. Most hand-sanitizers usually organic-based are also known to kill pathogen up to 99.9 % when effectively rubbed on the hand, but the absorption potential through human skin to some vital organs such as the kidney and liver could result to toxicities such as nephrotoxicity, cirrhosis among others (Kramer et al., 2007[48]; Choi et al., 2017[22]; Chan and Chan, 2018[20]). 

Since HH is of paramount importance to the prevention of the spread of virus and other germs, the use of safe hand rub, soap, or sanitizers is imperative. Due to associated toxicities and side effects, some hand rubs, soaps and sanitizers contain some chemical compounds which may only be most appropriate for use on non-skin surfaces such as the surfaces of furniture, door handles, walls, phones, glass, toilet zincs, metal tools, vehicles exteriors, electronic gadgets, hospital equipment among others and not on human skin. 

In order to underscore the relevance of alcohol in disinfection of non-skin objects, a study reported that a single cleaning with an alcohol wipe proved higher potency than the alcohol-based hand rub in decontamination of stethoscopes used in hospitals (Mehta et al., 2010[61]). Other studies also showed that use of hand sanitizers used outside hospital environment do not show superior benefit to soap (Oughton et al., 2009[68]; CDC, 2020[19]). Hence, washing with soap and warm water with good HH may adequately suffice for homes and as well eliminate exposure to hazardous chemicals and consecutive release to the environment (Wolf et al., 2018[99]). The CDC and some other reports indicated that besides preserving good environmental flora, washing with soap and water produces better or equivalent results compared to ABHS sanitizers at removing or killing certain types of virus such as the noroviruses and other germs (Grayson et al., 2009[38]; Blaney et al., 2011[11]; Bondurant et al., 2019[13]; CDC, 2020[18]).

### Impact of excessive use of hand sanitizers on the environment

Hand sanitizers usually end-up being deposited in high concentrations of the constituting chemical residues (contaminants) in the environmental soil and water bodies. High concentration of these chemicals released in the environment could trigger multi-drug resistance (Westgate et al., 2016[94]). Antimicrobial resistance is obviously a major public health concern globally. The World Health Organization (WHO) reported that about 700,000 people died due to antimicrobial-resistant infections every year (WHO, 2019[96]). It is further predicted that the death due to antimicrobial-resistant infections could reach 10 million annually by 2050 if no concrete action is taken now (WHO, 2019[96]).

### Preference for the use of ordinary soap or liquid soap for hand sanitizing 

Experiment has revealed that washing with ordinary soap solution is effective at killing and eliminating the virus and other germs in the hand. The fatty outer surface of the virus is denatured by the soap molecule and that process leads to the destruction of the virus which is washed away by water. It is worth mentioning that hand washing with soap solution and rinsing with water (preferably warm water) will kill and eliminate the germs from the surface, using of ABHS will kill the organism without eliminating them from the surface. The use of soap and water must be a preferred choice when the hand is visibly or grossly contaminated. ABHS also do not remove pesticide from the hand (CDC, 2020[18]). The use of ABHS in such instances could make the hand so messy and unpleasant. In fact, the Food and Drug Administration (FDA) of the United States already banned the use of triclosan in antibacterial soaps (IARC, 1998[44]; Halden, 2014[39]).

### Modern natural “Green” hand sanitizers as preferred alternative to ABHS

Recent advances in the cosmetic industry have led to the production of safe hand sanitizers obtained via green process. This involved the incorporation of safe natural antiseptic agents into soaps, hand rubs or NABHS in place of the synthetic ones that are associated with multiple side effects. Natural products such as plant extracts, exudates, purified isolates and essential oils have been proposed as germ-killers in hand sanitizers, soaps and other cosmetics and body care products (Ningsih et al., 2017[66]; Hartatik, 2014[40]; Lateh, 2015[52]; Surini et al., 2018[86]; Zubair et al., 2018[103][104]; Atolani et al., 2019[6][5]; 2020[4]). Natural products such as coconut oil have been used to produce hand sanitizers with enhanced cosmetic properties (Tran et al., 2019[90]). Such green alternative will preserve the natural environment and the skin of people (Atolani et al., 2016[7]).

### Recommendations for use of alcohol-based hand sanitizers and soaps

Following the multiple side-effects associated with the use of ABHS and some liquid soaps due to the “unsafe” active agents in them, the following recommendations have become inevitable:

1. Manufacturers and Health care providers should advocate the potential dangers associated with abuse, overuse and misuse of ABHS and antibacterial active soaps.
2. The habitual use of ABHS must be deliberately avoided. As much as it is possible, its use should be occasional when other options such as soap and warm water are not available. This would impact safety of both users and the environment.
3. Users should only purchase small amount of ABHS at a time to avoid overt addiction and abuse due to the surplus.
4. The use of hand sanitizer should be limited to only the finger and wrist region as absorption via the arm and other body parts could be increased thereby raising blood alcohol concentration within a short time.
5. When the use of ABHS is unavoidable, users should ensure use of low concentration alcohol at an average of 60-70 % as higher concentrations could be more deleterious.
6. The ABHS should be kept far away from heat, flame, spark-source or any oxidizing agents to avoid fire incidence since they are flammable.
7. Children should not be allowed direct access to ABHS as the fatality of accidental ingestion in children is worse and incidences are on the rise.
8. In case of liquid soaps; soaps with triclosan or triclocarban and other questionable safety profile should be avoided.
9. There should be incorporation of skin protecting emollient in ABHS and liquid soap in order to prevent after-use skin dryness, skin breaking/cracking as well as reduce alcohol penetration through the skin.
10. General cleanliness and good hygiene is imperative to keep microbial infections at bay. Consistent tidiness will minimize the need for repetitive use of liquid soaps and ABHS.

### Conclusions

The enormous release of adulterated and uncontrolled infection-preventing household products such as the antibacterial active liquid soap and ABHS in the global markets have several potential adverse health effects on human such as dehydrated skin, irritation, poisoning, and cancer among others. Thus, the careful adoption of hand washing with selected safe liquid soap should be encouraged by all. Improvement in hand hygiene is akin to the containment of the spread of germs, including the ravaging viral infection, COVID-19. However, when the use of hand sanitizer is inevitable, consumers should be cautious of the chemical constituents as well as the concentration of each constituent. These measures are important to the prevention of avoidable implications such as unintentional or deliberate ingestion or chemical absorption through the skin which could lead to incidences such as irreversible blindness, depression, intoxication, liver cirrhosis, acidosis, headache, central nervous system depression, seizure, hypoglycemia, coma and death. While preventing infections such as the COVID-19 and other microbial-induced infections, slow and systematic death should be meticulously avoided. Otherwise, the prevention mode could lead to higher fatality than the infection being avoided.

## Notes

Olubunmi Atolani and Oluyomi Stephen Adeyemi (Department of Biochemistry, Medicinal Biochemistry, Infectious Diseases, Nanomedicine & Toxicology Laboratory, Landmark University, PMB 1001, Omu-Aran 251101, Kwara State, Nigeria; E-mail: yomibowa@yahoo.com) equally contributed as corresponding author. 

## Acknowledgements

Authors appreciate Ton Duc Thang University, Ho Chi Minh City as well as the University of Ilorin, Ilorin, Nigeria.
